# Effect of Persian traditional medicine based herbal vaginal gel in vaginal atrophy

**DOI:** 10.1016/j.jaim.2025.101193

**Published:** 2026-04-01

**Authors:** Elahe Naderi-afshar, Mahboubeh Valiani, Mojgan Tansaz, Shirin Fahimi, Zahra Allameh, Maryam Ranjbar, Mohammad Mazaheri

**Affiliations:** a Department of Persian Medicine, Faculty of Medicine, Isfahan University of Medical Sciences, Isfahan, Iran; b Nursing and Midwifery Care Research Center, Faculty of Nursing and Midwifery, Isfahan University of Medical Sciences, Isfahan, Iran; c Department of Traditional Medicine, School of Traditional Medicine, Shahid Beheshti University of Medical Sciences, Tehran, Iran; d Traditional Medicine and Materia Medica Research Center and Department of Traditional Pharmacy, School of Traditional Medicine, Shahid Beheshti University of Medical Sciences, Tehran, Iran; e Department of Traditional Pharmacy and Persian Medicine, Faculty of Pharmacy and Pharmaceutical Sciences, Tehran Medical Sciences, Islamic Azad University, Tehran, Iran; f Department of Obstetrics and Gynecology, Medical School, Isfahan University of Medical Sciences, Isfahan, Iran

**Keywords:** Vaginal atrophy, Auriculotherapy, Matricaria Chamomilla L., Rosa damascene Herrm, Alcea digitate Alef., Malva Sylvestris L., Persian medicine

## Abstract

**Background:**

Vaginal atrophy is a common condition in postmenopausal women due to low estrogen causing dryness, irritation, infections, and painful intercourse. Treatments include non-hormonal (moisturizers, lubricants) and hormonal options (vaginal estrogens, phytoestrogens) Moreover, Traditional Persian medicine suggests plants as a potential remedy for vaginal atrophy. Auriculotherapy is also gaining popularity as a non-invasive and efficient treatment option.

**Objective:**

To evaluate the effect of a vaginal herbal gel and auriculotherapy compared to a placebo on vaginal atrophy of postmenopausal women.

**Materials and methods:**

In a single-blind, randomized trial, 90 menopausal women with vaginal atrophy symptoms were divided into Herbal gel, Auriculotherapy, and placebo groups (30 each). The study measured subjective symptoms (SSS-VVA), sexual function (FSFI), and quality of life (MENQOL) before, at four-, and eight-weeks post intervention.

**Results:**

Comparison of SSS-VVA showed significant improvement in vaginal itching, dryness, and pain in all groups compared to baseline. The herbal gel group demonstrated a more significant reduction in atrophic vaginitis indicators compared to placebo (P < 0.001) and greater decrease in vaginal dryness than auriculotherapy (P = 0.006). Additionally, both herbal gel and auriculotherapy groups exhibited significant improvements in FSFI, with desire (p = 0.038), orgasm (p < 0.001), and satisfaction (p = 0.004) showing greater improvement with herbal gel than auriculotherapy. Regarding MENQOL, both interventions improved patients' condition, with auriculotherapy showing overall greater improvement than herbal gel.

**Conclusion:**

Both interventions effectively reduced symptoms and improved quality of life, but the herbal gel outperformed auriculotherapy in reducing dryness and enhancing sexual function.

## 1 Introduction

Menopause represents a significant event for middle-aged women, marking a phase where they spend about one-third of their lifespan [1]. Vaginal atrophy, often due to estrogen deficiency post-menopause, leads to symptoms like dryness, irritation, itching, and dyspareunia, thereby negatively impacting sexual function and overall quality of life [2]. It is estimated that a significant proportion of postmenopausal women, ranging from 36 % to almost 90 %, experience symptoms associated with vulvovaginal atrophy (VVA). Several studies have reported symptoms related to the urogenital system during and after menopause [[3], [4], [5], [6], [7]]. Only approximately 25 % of women with symptoms of VVA seek medical assistance, due to various reasons. Moreover, women's attitudes towards this issue appear to differ across countries, likely influenced by the quality and accessibility of healthcare services [8]. This lack of reporting can result in underdiagnoses and under treatment of the condition, despite the significant impact that VVA symptoms can have on daily activities, sexual function, interpersonal relationships, and overall quality of life for postmenopausal women [[9], [10], [11]]. However, further research is needed to understand the full extent of these symptoms on postmenopausal women's quality of life [12].

Symptoms of vaginal atrophy can be managed through various treatments. Non-hormonal options like moisturizers and lubricants are recommended as first-line treatments for mild cases [13,14]. It is important to note that while these interventions may increase sexual activity and improve sexual function, they cannot reverse atrophic changes in the vagina. For moderate to severe cases of vulvovaginal atrophy that do not respond to non-hormonal interventions, vaginal estrogens containing conjugated estrogens or estradiol are frequently used as a secondary option [[15], [16], [17]]. Before starting estrogen therapy, it is essential to ensure no contraindications exist, especially a history of estrogen-dependent tumors. While side effects of vaginal estrogen therapy are rare, some patients may experience irritation, bleeding, or breast tenderness [18].

Due to concerns about hormone therapy, non-hormonal treatments like lubricants and moisturizers are considered alternatives [[19], [20], [21]]. Herbal treatments are increasingly used due to fewer side effects and greater acceptance [22]. A significant majority of people (65–80 %) worldwide rely on herbal products for treatment [23]. Additionally, The WHO considers complementary medicine a way to alleviate menopausal symptom [24].

According to traditional Persian medicine, a formula consisting of extracts from *Matricaria Chamomilla* L.*, Rosa damascene Herrm., Alcea digitate Alef., and Malva Sylvestris* L. is suggested as a potential treatment for vaginal dryness [25]. Based on recent studies, these products are considered safe when used occasionally or in combination [26,27]. The Matricaria Chamomilla is known for therapeutic properties such as phytoestrogens, analgesic, anti-inflammatory, antimicrobial, antispasmodic, sedative, and its wound-healing effects [28]. Likewise, the Alcea digitate plant is recognized for its medicinal qualities, including antimicrobial, anti-inflammatory, immune system-modulating, and emollient properties [29]. Additionally, Malva Silvestris utilized in researchs for its mucilage, antibacterial, antifungal, and antioxidant characteristics [30]. Lastly, the Rosa damascene plant is attributed with multiple beneficial features like analgesic, anti-microbial, antioxidant, anti-inflammatory, laxative, and softening effects [31].

In recent years, acupuncture and ear acupuncture (auriculotherapy), have gained popularity as safe and effective methods. Additionally, ear acupuncture has gained attention as a treatment that stimulates the external surface of the ear to alleviate pathological conditions in other parts of the body. This technique can be performed using various methods such as finger pressure, electrical stimulation, laser, needles, magnetic seeds, and plant seeds [[32], [33], [34], [35], [36], [37], [38], [39]].

However, with the increasing global interest in accessible traditional and complementary medicine, we aimed to explore auriculotherapy's potential benefits. Given the widespread use of body acupuncture and auriculotherapy's ease of use, it offers a practical TCM approach. Studies suggest auriculotherapy can modulate the autonomic nervous, potentially improving blood flow. While direct hormonal evidence is limited, acupuncture may influence hormonal balance [40]. Preliminary data from our work also showed promise. This rigorously designed study is exploratory, aiming to evaluate the impact of a vaginal herbal gel and auriculotherapy compared to a placebo in a Randomized Clinical Trial among menopausal women, assessing changes in atrophy symptoms, quality of life, and sexual function.

## 2 Materials and methods

### 2.1 Study design and participants

This was an eight-week, single-blind, randomized, clinical trial on menopausal women (>12 months after the last menstrual cycle) aged 45 to 65 referring to obstetric-gynecologist clinics associated with Isfahan University of Medical Sciences from May 2022 to May 2023. The study was approved by the ethics committee of Isfahan University of Medical Sciences (IR.MUI.MED.REC.1400.699) and registered in the Iranian Registry of Clinical Trials (IRCT20211013052757N1). All volunteers provided written informed consent before study entry.

One hundred and five participants were selected for this study using the formula below considering a type I error of 0.05, statistical power of 80 %, and information from Bosak et al.'s research, considering 15 % probable dropout [27].

$$n=\delta_{\alpha ,\beta }{\left[\sum_{i=1}^{r}\sum_{j=1}^{c}\frac{{\left(p_{ij-}p_{i}p_{j}\right)}^{2}}{p_{i}p_{j}}\right]}^{-1}$$

### 2.2 Random allocation and concealment

Volunteers were assigned to three groups (vaginal gel, placebo, and auriculotherapy) through a random allocation list generated by R4.0.2 software. The pharmacist coded the herbal gel and placebo. The statistician remained blinded to group allocation. To ensure randomization, researchers employed the permutation block method, randomly dividing samples into three groups of 35 participants each, with blocks of six people. Patient allocation occurred after obtaining informed consent.

### 2.3 Inclusion and exclusion criteria

Postmenopausal women who met the following inclusion criteria were enrolled. 1) Being married. 2) The presence of symptoms of vaginal atrophy (dyspareunia, burning, itching, dryness) confirmed by an obstetric-gynecologist. 3) Normal Pap smear in the past year; Patients with the following criteria were not included. 1) Having breast or uterine cancer. 2) Abnormal vaginal bleeding. 3) Anomalies within the vaginal system. 4) Vaginal infection requiring treatment. 5) Taking oral hormonal drugs (such as OCP, estrogen, progesterone, etc.) eight weeks before treatment. 6) Use of local hormonal creams (Stromarin, Premarin, etc.) or topical lubricants during the last four weeks. 7) Previous history of allergy to studied medicinal plants. The criteria for withdrawal from the study were: 1) Occurrence of drug sensitivity. 2) Taking hormonal drugs (such as OCP). 3) Failure to employ auriculotherapy for two consecutive sessions or three alternating sessions. 4) Not using vaginal gel for five consecutive days or ten alternate days. 5) Unwillingness to continue the project at any time and for any reason. 6) Vaginal infection that needs treatment (referral to a gynecologist).

### 2.4 Herbal gel preparation

**Fig. 1:**
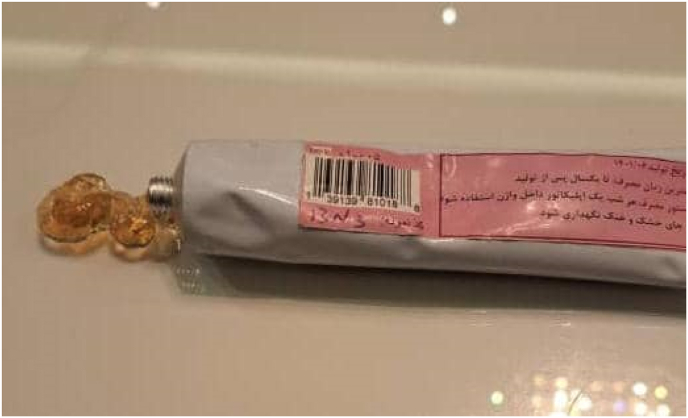
Herbal gel.

To prepare herbal vaginal gel, dried flowers of Matricaria Chamomilla L., Rosa damascene Herrm., Alcea digitate Alef., and Malva Sylvestris L. were purchased from Tehran herbal market. After identifying, Herbal Market Samples (HMS) of Rosa Damascena Herrm. (HMS No. 569), Alcea digitate Alef. (HMS No. 570), Malva Sylvestris L. (HMS No.571) and Matricaria Chamomilla L. (HMS No.572) were deposited at the Herbarium of Traditional Medicine and Materia Medica Research Center (TMRC), Shahid Beheshti University of Medical Sciences, Tehran, Iran. An aqueous extract of four plant materials (with equal proportions) was prepared using the decoction method (total of plant materials: water, 1:20). The herbal gel consists of 2.25 % dry extract of the mentioned plants in a base containing 1.75 % carbomer and microbial preservatives including methyl and propyl parabens (0.2 % and 0.06 %, respectively). Propylene glycol was used as a co-solvent in the formulation. The herbal formulation was satisfactory concerning its physical parameters and microbial contents. Moreover, product standardization was performed based on total polyphenols of plant components (120 mg/100g, based on pyrogallol). The placebo gel was formulated with the same physical properties as herbal gel by using the same ingredients except for the plant extract. Caramel color (88K, MAPTRAL-15001D, MAP-DIANA Company, England) was applied to match the color of the placebo gel with that of the medicine. Both the herbal and placebo gels were formulated and prepared in the Traditional Medicine and Materia Medica Research Center, Shahid Beheshti University of Medical Sciences, Tehran, Iran. The prepared gels were filled into identical 50-g tubes and provided to the patient with an applicator (Fig. 1).

### 2.5 Measured variables

Demographic data, including age, age of menopause, Menarche age, BMI, Duration of menopause, educational level, and smoking status, were collected at the study baseline. Participants were examined by an obstetric-gynecologist (OB/GYN) specialist to evaluate signs and symptoms of atrophic vaginitis. The researcher completed the baseline section of the study data collection form for each patient, which included the evaluation form of subjective symptoms of vulvovaginal atrophy (SSS-VVA), the questionnaire of the Female Sexual Function Index (FSFI), and the questionnaire for evaluating the Menopause-Specific quality of life (MENQOL). It should be noted that the validity and reliability of the FSFI and MENQOL questionnaires of postmenopausal women have been confirmed.

SSS-VVA assesses the severity of itching, burning, vaginal dryness, and painful intercourse, with each symptom scored on a scale from 0 (absent) to 4 (very severe). Individuals with a score of 3 or higher were included in the study.

FSFI measures women's sexual performance with 19 questions in 6 areas including sexual desire, arousal, lubrication, orgasm, satisfaction, and pain. The full score ranges from 2 to 36.

The questions of the quality-of-life questionnaire are organized into four dimensions: vasomotor, psychosocial, physical, and sexual disorders, comprising a total of 26 questions.

### 2.6 Intervention and measurements

In the groups that utilized vaginal gel (herbal and placebo), patients were trained and prescribed to use three tubes of either herbal or placebo vaginal gel. These tubes were 50 g in size and identical in shape. The patients were provided with a drug consumption checklist to ensure regular compliance with using the vaginal gel. For the first four weeks of treatment, patients were advised to use the medication nightly before bed time. This involved using a 5-g applicator inside the vagina and remaining in a reclined position for several hours until the medicine was absorbed. At the end of the fourth week, the FSFI, SSS-VVA, and MENQOL questionnaires, were completed by a researcher for each patient, as well as a medication side effect registration form. Subsequently, two additional tubes of the vaginal gel were provided to the patients for use during the following four weeks. During the second four weeks of treatment, patients were instructed to use the vaginal gel every other night before bed time. The procedure was similar to that of the initial four weeks. To ensure regular compliance with the treatment regimen and to address any potential queries, the researcher maintained weekly phone communications with the patients. At the end of the eight-week treatment period, all forms were filled out once again.

In the auriculotherapy group, the patients come once a week for auriculotherapy from the beginning of the study. During each visit, the points related to vaginal atrophy in the ear are identified, and Vaccaria seed is placed on the corresponding points. In postmenopausal women, estrogen is produced through the peripheral aromatization of ovarian and adrenal androgens. Therefore, auriculotherapy stimulates the points responsible for androgen production in the ovaries and adrenals, as well as the points in the pelvis, vagina, and uterus to activate estrogen receptors [40]. The protocol included the following ear points: ovary, endocrine, external genitalia, Lung-1 on the left ear, and master shoulder, thalamic, and pelvic points on the right ear. The patient is then instructed to press each side for 1 min every hour, except during sleep. Taking a shower while having ear seeds is not an issue. Forms and questionnaires were completed in the auriculotherapy group at the beginning of the intervention, as well as at the end of the fourth and eighth weeks.

### 2.7 Statistical analysis

To evaluate the normality of the distribution of quantitative variables, graphical methods were used, and the description of qualitative variables using frequency and percentage. The description of quantitative variables was reported using the mean and standard deviation. Comparison between groups at any point in time was done using the analysis of variance method (ANOVA). Comparing the mean changes in each of the groups at the beginning of the study and the eighth week of follow-up was done with a paired *t*-test. Repeated measure ANCOVA was used to compare the effectiveness of each treatment with each other with adjustment on the base value. P-value<0.05 was considered significant. All data were analyzed by SPSS software (SPSS Statistics for Windows, version 26.0).

## 3 Results

**Fig. 2:**
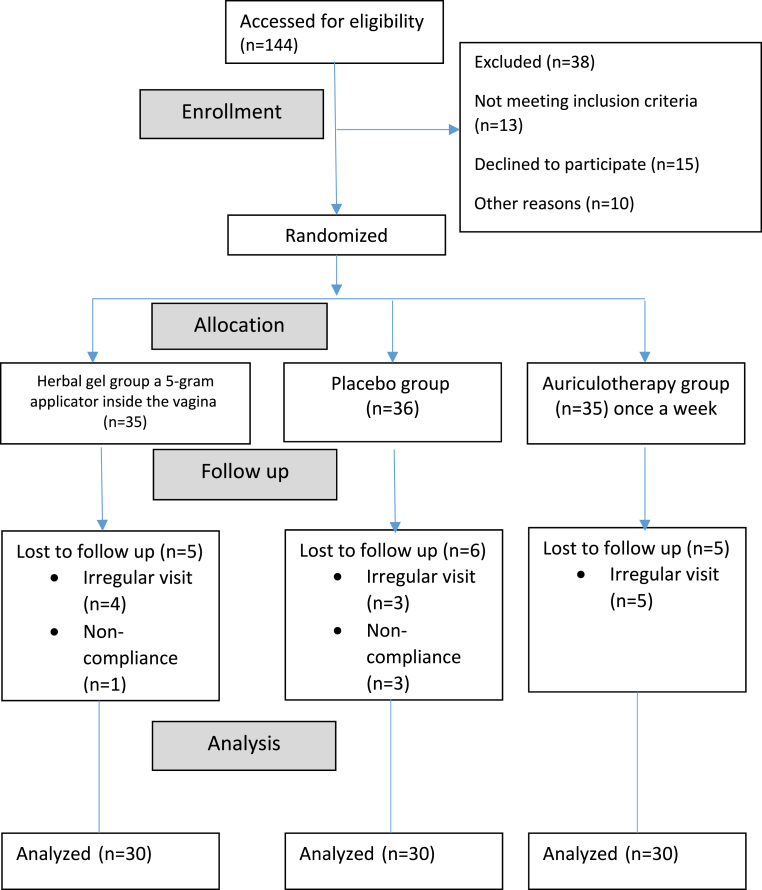
Study flowchart.

The study begins with a total of 106 eligible participants out of 144. Ultimately, 90 women (30 in each group) completed the trial (Fig. 2).

### 3.1 Demographic and baseline data

The analysis of demographic data revealed no significant differences in the mean age, average BMI indices, menarche age, age at onset of menopause, or duration of menopause across the three groups. Further, the study of variables related to people's lifestyle, including smoking status and education, did not reveal any significant differences (Table 1).

**Table 1:** Demographic characteristics and lifestyle-dependent variables of patients.

	Herbal gel (n = 30)	Auriculotherapy (n = 30)	Placebo (n = 30)	P-value
**Smoking**				0.656
yes	6(20.0)	9(30.0)	7(23.3)	
no	24(80.0)	21(70.0)	23(76.7)	
**Number of deliveries**				0.924
1	1(3.3)	1(3.3)	1(3.3)	
2	6(20.0)	9(30.0)	7(23.3)	
3	8(26.7)	10(33.3)	10(33.3)	
≥4	15(50.0)	10(33.3)	12(40.0)	
**Education**				0.967
non-literate	4(13.3)	2(6.7)	3(10.0)	
High school degree	10(33.3)	11(36.7)	9(30.0)	
Diploma	11(36.7)	13(43.3)	14(46.7)	
Bachelor's degree or higher	5(16.7)	4(13.3)	4(13.3)	

**Age (year)**	56.63 ± 4.53	55.53 ± 3.76	56.47 ± 4.53	0.566
**Weight (kg)**	61.77 ± 8.19	59.30 ± 8.89	59.57 ± 8.92	0.485
**Height (m)**	1.63 ± 0.05	1.63 ± 0.06	1.63 ± 0.05	0.920
**BMI (kg/m2)**	23.30 ± 3.40	22.45 ± 3.13	22.47 ± 3.49	0.535
**Menarche age (year)**	12.40 ± 1.25	13.07 ± 0.78	12.73 ± 1.11	0.059
**Menopause age (year)**	49.60 ± 3.05	50.53 ± 2.92	50.17 ± 3.00	0.479
**Duration of menopause (year)**	3.50 ± 1.72	3.83 ± 2.21	3.33 ± 1.79	0.592

N(%) for qualitative variables and mean ± SD for quantitative variables.

#### 3.1.1 Group analysis

Comparison of baseline SSS-VVA features between the three groups showed that the indicators of itching, dryness, and pain improved significantly during the study (P1 in Table 2); however, the burning index did not change significantly over time in the placebo group (P = 0.096; Table 2). Additionally, the Herbal gel group had a significant reduction in all indicators compared to the placebo group (P∗∗∗ in Table 2). Some SSS-VVA indexes were not statistically different between Herbal and Auriculotherapy groups such as Itching (P = 0.121) burning (p = 0.898), and pain (p = 0.735). As shown in Table 2 the vaginal dryness in the Herbal gel group had a greater reduction compared to Auriculotherapy (P = 0.006).

**Table 2:** SSS-VVA feature indexes in Study Arms Before Intervention and After Follow-Ups.

						Repeated measure ANCOVA		
		**Herbal gel**	**Auriculotherapy**	**Placebo**	**P2**	**P ∗**	**P ∗∗**	**P ∗∗∗**
itching								
	Baseline	1.80 ± 1.06	1.40 ± 0.89	1.90 ± 0.92	0.110	<0.001	0.121	<0.001
	Week 4	0.80 ± 0.71	0.83 ± 0.75	1.93 ± 0.91	<0.001			
	Week 8	0.57 ± 0.68	0.40 ± 0.62	1.67 ± 0.76	<0.001			
	**P1**	<0.001	<0.001	0.006				
burning								
	Baseline	1.70 ± 1.09	1.70 ± 0.70	1.37 ± 0.81	0.245	<0.001	0.898	<0.001
	Week 4	0.77 ± 0.63	0.97 ± 0.56	1.50 ± 0.73	<0.001			
	Week 8	0.57 ± 0.50	0.40 ± 0.67	1.20 ± 0.85	<0.001			
	**P1**	<0.001	<0.001	0.096				
dryness								
	Baseline	3.43 ± 0.50	2.87 ± 0.86	2.93 ± 1.20	0.033	<0.001	0.006	<0.001
	Week 4	1.00 ± 0.74	1.20 ± 0.66	2.83 ± 1.23	<0.001			
	Week 8	0.67 ± 0.55	0.63 ± 0.72	2.57 ± 1.30	<0.001			
	**P1**	<0.001	<0.001	<0.001				
pain								
	Baseline	3.07 ± 0.83	2.87 ± 1.07	2.17 ± 0.75	<0.001	<0.001	0.735	<0.001
	Week 4	1.07 ± 0.78	1.00 ± 1.05	2.17 ± 0.65	<0.001			
	Week 8	0.67 ± 0.61	0.67 ± 0.99	1.70 ± 0.99	<0.001			
	**P1**	<0.001	<0.001	<0.001				

SSS-VVA: subjective symptoms of vulvovaginal atrophy.

P1: comparing post-intervention and baseline values in each group using paired samples *t*-test.

P2: between-group comparison at each time point using ANOVA.

P ∗: between group comparison adjusted for bassline value using repeated measure ANCOVA.

P∗∗: "Herbal gel vs. Auriculotherapy" adjusted for baseline value using repeated measure ANCOVA.

P∗∗∗: "Herbal gel vs. Placebo" adjusted for baseline value using repeated measure ANCOVA.

**Fig. 3:**
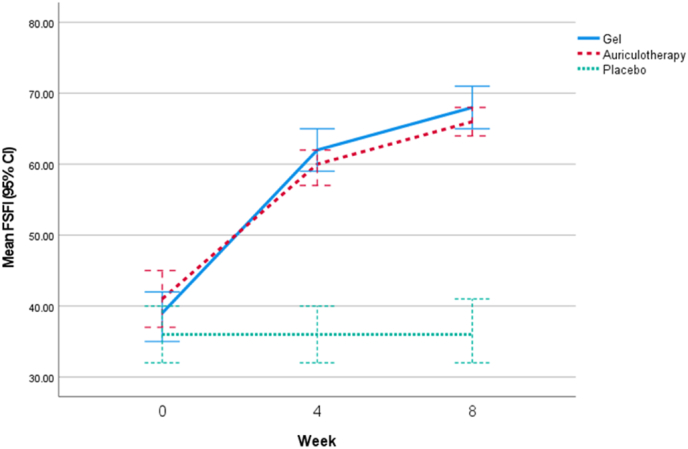
Changes in mean (95 % CI) total FSFI score in the three groups over time.

Comparisons of the FSFI indexes before Intervention and after follow-ups, for each of the three groups show that the average of indicators improved significantly in the herbal gel and auriculotherapy groups, but there were no significant changes observed in the placebo (P1 in Table 3).In the Herbal gel group, all areas of FSFI increased significantly in comparison to the Placebo (P∗∗∗ in Table 3) and in comparison to Auriculotherapy the score of Desire (P = 0.038), Orgasm (P < 0.001), satisfaction (P = 0.004), and FSFI total score (P = 0.005) was significantly more improved in the Herbal gel group. Nevertheless, there was no significant difference in scores between the group treated with herbal gel and auriculotherapy in the areas of Arousal (P = 0.647), Lubrication (P = 0.537), and Pain (P = 0.074). By comparing the mean changes of FSFI score in the Herbal gel group versus Placebo (6.4 ± 29.23 vs 1.33 ± 0.50) a standardized effect size value of 6.09 (95 % CI: 4.884, 7.29) was calculated, indicating a substantial effect size[Fig. 3].

**Table 3:** Total score of FSFI and its components between study arms Before Intervention and After Follow-Ups.

						Repeated measure ANCOVA		
		**Herbal gel**	**Auriculotherapy**	**Placebo**	**P2**	**P ∗**	**P ∗∗**	**P ∗∗∗**
Desire								
	Baseline	3.50 ± 1.07	3.87 ± 1.20	3.70 ± 1.21	0.475	<0.001	0.038	<0.001
	4 week	5.70 ± 1.18	5.70 ± 0.84	3.80 ± 1.35	<0.001			
	8 week	6.53 ± 1.20	6.13 ± 0.78	3.77 ± 1.30	<0.001			
	**P1**	<0.001	<0.001	0.423				
Arousal								
	Baseline	7.90 ± 2.40	8.50 ± 3.37	7.07 ± 2.64	0.15	<0.001	0.647	<0.001
	4 week	12.20 ± 2.41	12.33 ± 2.84	7.43 ± 2.85	<0.001			
	8 week	13.20 ± 2.20	13.50 ± 2.54	7.40 ± 2.85	<0.001			
	**P1**	<0.001	<0.001	0.067				
Lubrication								
	Baseline	9.97 ± 1.59	10.17 ± 2.13	9.43 ± 2.16	0.337	<0.001	0.358	<0.001
	4 week	13.17 ± 1.56	13.17 ± 1.44	9.33 ± 1.88	<0.001			
	8 week	14.23 ± 1.19	13.97 ± 1.03	9.50 ± 2.03	<0.001			
	**P1**	<0.001	<0.001	0.645				
Orgasm								
	Baseline	5.97 ± 2.08	6.70 ± 3.37	5.10 ± 2.23	0.067	0.002	<0.001	<0.001
	4 week	10.10 ± 1.60	9.33 ± 2.23	5.17 ± 2.31	<0.001			
	8 week	10.73 ± 1.80	10.20 ± 1.61	5.17 ± 2.42	<0.001			
	**P1**	<0.001	<0.001	0.601				
Satisfaction								
	Baseline	6.00 ± 1.89	5.70 ± 1.74	5.43 ± 2.13	0.526	<0.001	0.004	<0.001
	4 week	10.60 ± 1.33	9.80 ± 1.47	5.40 ± 2.30	<0.001			
	8 week	12.17 ± 1.78	10.90 ± 1.21	5.50 ± 2.36	<0.001			
	**P1**	<0.001	<0.001	0.573				
Pain								
	Baseline	5.97 ± 2.11	6.43 ± 2.82	5.53 ± 2.54	0.385	<0.001	0.074	<0.001
	4 week	10.70 ± 1.84	9.73 ± 1.51	5.37 ± 2.37	<0.001			
	8 week	11.67 ± 2.02	12.00 ± 1.08	5.43 ± 2.34	<0.001			
	**P1**	<0.001	<0.001	0.448				
FSFI								
	Baseline	39.30 ± 9.31	41.37 ± 11.23	36.27 ± 11.25	0.181	<0.001	0.005	<0.001
	4 week	62.47 ± 7.77	60.07 ± 7.00	36.50 ± 11.42	<0.001			
	8 week	68.53 ± 8.47	66.70 ± 5.15	36.77 ± 11.67	<0.001			
	**P1**	<0.001	<0.001	0.05				

FSFI: Female Sexual Function Index.

P1: comparing post-intervention and baseline values in each group using paired samples *t*-test.

P2: between-group comparison at each time point using ANOVA.

P ∗: between group comparison adjusted for bassline value using repeated measure ANCOVA.

P∗∗: "Herbal gel vs. Auriculotherapy" adjusted for baseline value using repeated measure ANCOVA.

P∗∗∗: "Herbal gel vs. Placebo" adjusted for baseline value using repeated measure ANCOVA.

**Fig. 4:**
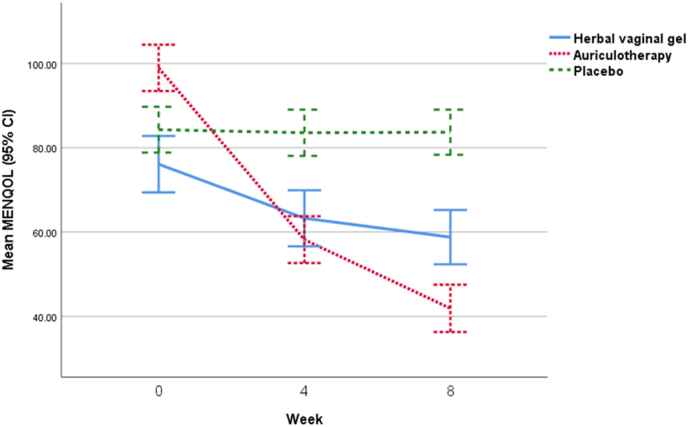
Changes in mean (95 % CI) total MENQOL score in the three groups over time.

Table 4 displays the results of average changes in the MENQOL questionnaire and its related areas before and after the intervention, at the 4- and 8-week follow-up periods, for all three groups. The findings reveal that patients' condition improved over time in all areas of MENQOL in the Herbal gel and Auriculotherapy intervention groups, but no significant changes were observed in the placebo gel group (P1 in Table 4). Using repeated analysis of variance to compare the effectiveness of each treatment, the group treated with herbal gel demonstrated a greater decrease in all MENQOL domains, except for the Psychosocial index (P = 0.088), compared to Placebo. Additionally, the total MENQOL score in the Herbal gel group had a greater decrease than the Placebo (p < 0.001). In contrast, patients in the Auriculotherapy group showed a greater decrease in all indicators, except sexual (p = 0.113), compared to the Herbal gel treatment group (P∗∗ in Table 4). Essentially, the Auriculotherapy group experienced more improvement in quality of life than the Herbal gel group. By comparing the mean changes in the MENQOL score in the Herbal gel group versus the Placebo (12.44 ± 17.3 vs 3.58 ± 0.6), a standardized effect size of −1.82 (95 % CI: 2.42, −1.22) was calculated. This indicates a large effect size, suggesting that the use of Herbal gel may not be as effective as Auriculotherapy in improving the quality of life for menopausal women [Fig. 4].

**Table 4:** Total score of MENQOL and its components between study arms Before Intervention and After Follow-Ups.

						Repeated measure ANCOVA		
		**Herbal gel**	**Auriculotherapy**	**Placebo**	**P2**	**P ∗**	**P ∗∗**	**P ∗∗∗**
Vas motor								
	Baseline	5.03 ± 2.70	10.60 ± 2.27	8.27 ± 2.85	<0.001	<0.001	<0.001	<0.001
	4 week	4.20 ± 1.94	5.53 ± 2.32	8.30 ± 2.93	<0.001			
	8 week	3.70 ± 1.88	3.77 ± 2.43	8.10 ± 2.83	<0.001			
	**P1**	0.001	<0.001	0.169				
Psychosocial								
	Baseline	19.47 ± 8.52	25.73 ± 6.62	19.13 ± 7.97	0.002	<0.001	<0.001	0.088
	4 week	18.27 ± 8.62	14.50 ± 4.07	19.07 ± 8.17	0.038			
	8 week	17.67 ± 8.24	9.40 ± 4.31	19.20 ± 8.04	0			
	**P1**	0.028	<0.001	0.829				
Physical								
	Baseline	38.07 ± 9.71	48.57 ± 9.50	43.73 ± 9.16	<0.001	<0.001	<0.001	0.001
	4 week	33.80 ± 9.80	29.93 ± 9.09	42.70 ± 8.79	<0.001			
	8 week	33.07 ± 9.70	23.50 ± 8.50	42.83 ± 8.61	<0.001			
	**P1**	<0.001	<0.001	0.135				
Sexual								
	Baseline	13.53 ± 2.30	14.07 ± 2.60	13.17 ± 3.31	0.452	<0.001	0.113	<0.001
	4 week	7.00 ± 2.84	8.23 ± 2.22	13.50 ± 3.01	<0.001			
	8 week	4.37 ± 1.52	5.23 ± 2.70	13.57 ± 2.93	<0.001			
	**P1**	<0.001	<0.001	0.11				
MENQOL								
	Baseline	76.10 ± 17.95	98.97 ± 14.74	84.30 ± 14.56	<0.001	<0.001	<0.001	<0.001
	4 week	63.27 ± 17.80	58.20 ± 14.84	83.57 ± 14.70	<0.001			
	8 week	58.80 ± 17.24	41.90 ± 15.03	83.70 ± 14.33	<0.001			
	**P1**	<0.001	<0.001	0.366				

MENQOL: Menopause-Specific quality of life.

P1: comparing post-intervention and baseline values in each group using paired samples *t*-test.

P2: between-group comparison at each time point using ANOVA.

P ∗: between group comparison adjusted for bassline value using repeated measure ANCOVA.

P∗∗: "Herbal gel vs. Auriculotherapy" adjusted for baseline value using repeated measure ANCOVA.

P∗∗∗: "Herbal gel vs. Placebo" adjusted for baseline value using repeated measure ANCOVA.

### 3.2 Adverse effects

A participant experiencing redness with herbal gel discontinued the study but showed improvement with calendula.

## 4 Discussion

This study shows that the use of herbal gel and auriculotherapy can enhance the signs and symptoms of vaginal atrophy in menopausal patients. These two methods can be suggested as non-hormonal complementary treatments with minimal risk of side effects.

Despite hormone therapy's high effectiveness and advantages [41], its usage has decreased in recent years due to the presence of side effects(19, 20). Hormonal treatments have adverse effects that include increased risk of cardiovascular events, thromboembolic diseases, breast cancer, stroke, uterine bleeding, disorders in lipid metabolism, endometrial hyperplasia, as well as various other side effects such as edema, breast pain, abdominal bloating, and headaches [42]. Introducing non-hormonal complementary treatments can be an effective solution for treating vaginal atrophy, as they are often more affordable and devoid of side effects.

In several articles, the effect of vaginal products containing phytoestrogens on vaginal atrophy has been investigated. These products are prepared from medicinal plants containing phytoestrogens such as Chamomile, Pueraria Mirifica, Liquorice, Fenugreek seeds, Flax seeds, Black cohosh, Fennel, Soy and Red Clover are useful in reducing the symptoms of vaginal atrophy in menopausal women. In a randomized clinical study conducted by Suwanvesh et al., the effectiveness of 12 weeks of Pueraria Mirifica vaginal gel and conjugated estrogen vaginal cream in treating postmenopausal women with vaginal atrophy was examined. The results proposed Pueraria Mirifica vaginal gel as a safe and effective treatment for vaginal atrophy [43]. In another double-blind clinical trial conducted by Sadeghi et al. on 70 menopausal women in Izeh, the effect of licorice vaginal cream 2 % significantly improved symptoms and signs of vaginal atrophy [44]. Mazalzadeh et al. concluded that the use of Fenugreek vaginal cream compared to placebo reduces painful intercourse and increases sexual desire in postmenopausal women [45]. Moreover, Asgharpour et al., in their systematic review of 12 clinical trials out of 747 articles, noted that herbal products obtained from seven medicinal plants containing phytoestrogens (Chamomile, Pueraria Mirifica, Liquorice, Flax seed, black Cohosh, fennel and red clover) have beneficial effects in reducing symptoms of vaginal atrophy in menopausal women [46]. However, in some studies, there was no evidence of the superiority of using herbal medicines compared to hormonal products [27].

The herbal gel used in this study consists of four plants that have been utilized for centuries in Traditional Persian Medicine due to their various healing abilities. Among the properties of these plants, phytoestrogens, analgesic, anti-inflammatory, antimicrobial, antispasmodic, sedative, and wound-healing effects can be named. These plants also have immune system-modulating, emollient, and antitussive properties, as well as mucilage, antibacterial, antifungal, and antioxidant characteristics. Moreover, they have laxative, and softening effects. The reduction of vaginal atrophy symptoms in the herbal gel group in our study can be attributed to the healing properties of these plants, particularly their moisturizing effect, ability to relieve inflammation [25,[28], [29], [30], [31]].

Chamomile **(***Matricaria chamomilla***)** is known for its various health benefits, including potential estrogenic activity. Several phytochemicals such as flavonoids, luteolin and coumarins are known to exhibit this kind of activity. Caffeic Acid, while not directly estrogenic, has antioxidant properties and may influence estrogen metabolism and signaling pathways [47,48]. Certain phytochemicals present in rose (*Rosa damascena*) may exhibit estrogenic activity such as flavonoids and essential oils as well as phenolic compounds such as gallic acid and ellagic acid. Moreover, tannins present in rose petals, may have weak estrogenic activity and can influence the bioavailability of other phytoestrogens, affecting overall hormonal balance [49,50]. In addition to the presence of flavonoids such as quercetin in hollyhock (*Alcea digitata*) and mallow **(***Malva sylvestris***)** which are responsible for the plants' estrogenic effects, the role of phenolic compounds such as caffeic acid and chlorogenic acid in the plants' estrogenic effects cannot be ignored. In addition, the plants' saponins and mucilages contents, can have tissue soothing and emollient effects and alliviate dryness problems. Some studies suggest that triterpenes and sterols found in *Alcea digitata* and *Malva sylvestris* may interact with estrogen receptors and exhibit estrogen-like activity. Their exact role in hormonal modulation requires further investigation [[51], [52], [53]]. The reduction of vaginal atrophy symptoms in the herbal gel group in our study can be attributed to the estrogenic properties of phytochemicals, particularly their moisturizing effect, ability to relieve inflammation [25,[28], [29], [30], [31]].

Our study revealed that the SSS-VVA indices, such as itching, dryness, and pain, improved significantly in the intervention groups. There are also studies suggesting the therapeutic effect of non-hormonal treatments for improving the subjective and objective symptoms of vulvovaginal atrophy.

In a prospective randomized cross-over trial conducted by Stute, P. et al., two non-hormonal vaginal drugs were investigated, and both topical products reliably and safely decreased the SSS-VVA [54].

A meta-analysis study of seven randomized controlled trials involving 342 participants, comparing the efficacy of acupuncture to controls or other interventions in reducing menopause-related symptoms. The analysis found that acupuncture can be an effective adjunctive treatment for menopause-related symptoms [55].

Our study also showed a significant improvement in FSFI indexes following the administration of Herbal gel and Auriculotherapy.

Another study supporting the use of Herbal gel reported a significant improvement in the sexual function index among postmenopausal women with sexual dysfunction who received Alcea digitate Alef vaginal suppositories [56].

We also found both groups of interventions improved patients' MENQOL score over time, but the auriculotherapy group showed greater improvement overall than the herbal gel group.

In recent decades, acupuncture and its associated techniques, such as ear acupuncture, have increasingly gained acceptance in Western medical culture. In fact, in 1997, the National Institutes of Health (NIH) released a statement endorsing auriculotherapy for the treatment of various medical conditions, including pain, nausea, and vomiting. The advantages of auriculotherapy are manifold. This method is relatively straightforward, non-invasive, safe, effective, cost-efficient, and fast-acting. Moreover, this technique is not only employed for the treatment of ailments but also serves as a useful tool for their diagnosis [37]. In a historical review and perspective on the impact of Acupuncture on U.S. Medicine conducted by Dominic P. Lu et al., the advantages and disadvantages of acupuncture compared to general anesthesia are stated. The disadvantages of this method include failure to respond to treatment, longer induction periods, acupuncture needle interference with surgical sites, and more bleeding may occur [57].

### 4.1 Study limitations

No post-study follow-up was done in this study.

## 5 Conclusion

This study showed that Herbal gel and Auriculotherapy can be safely used as a treatment option for vaginal atrophy in menopause patients. However, further studies with higher statistical power are still required to investigate the effect of this compound in patients who cannot tolerate estrogen.

## Data statement

The corresponding author will share SPSS file when it is requested by any researcher or reviewer.

## Conflict of interest

The authors declare no conflict of interest.
